# Chloroplast phylogenomic analysis provides insights into the evolution of *Paris liiana* sp. nov

**DOI:** 10.1080/23802359.2020.1867016

**Published:** 2021-02-08

**Authors:** Xin Guan, Qingshu Yang, Shuang Wang, Haizhu Zhang, Conglong Xia

**Affiliations:** a College of Pharmaceutical Science, Dali University, Dali, PR China; b Key Laboratory of Yunnan Provincial Higher Education Institutions for Development of Yunnan Daodi Medicinal Materials Resources, Dali, PR China

**Keywords:** *Paris liiana* sp. nov, genus *Paris*, complete chloroplast genome, phylogenetic analysis

## Abstract

*Paris liiana* sp. nov is a species of flowering herb of the genus *Paris* and widely distributed in the southwest of China. In this study, we sequenced the complete chloroplast (cp) genome of *P. liiana* sp. nov to investigate its phylogenetic relationship in genus *Paris*. The cp genome of *P. liiana* sp. nov was 163,860 bp in length, containing a large single-copy (LSC) region of 84,415 bp, a small single-copy (SSC) region of 12,947 bp, and a pair of inverted repeats (IRs) region of 33,249 bp. The overall GC content was 37.0%. The genome comprises of 135 genes, including 91 protein-coding genes, 37 tRNA genes, and 4 rRNA genes. Phylogenetic relationship analysis based on complete cp genome sequences exhibited that *P. liiana* sp. nov was most related to *P. polyphylla* var. *yunnanensis*.

*Paris polyphylla* var. *yunnanensis* is a perennial rhizomatous herb distributed in southwestern China and northern Myanmar (Li 1998). Studies have shown that *Paris* plant mainly contains steroidal saponins, triterpenoids, flavonoids and other chemical components, Steroidal saponins are the major active chemical constituents of *Paris* plants (Wang et al. 2015), including antitumor activity (Yan et al. 2009; Man et al. 2011), immuno-stimulating properties (Zhang et al. 2007), anthelmintic activity (Wang et al. 2010), antimicrobial activity (Qin et al. 2012), and protective effects on indomethacin-induced gastric mucosal lesions. It is also the material basis of some Chinese patent anticancer medicines, such as Gan-Fu-Le capsules, Bo-Er-Ning capsules, Lou-Lian capsules and so on (Wang et al. 2018). In the early stage of this study, we found some morphological differences among different samples of *P. polyphylla* var. *yunnanensis*, two phenotypes (“typical” and “high stem”). *Paris polyphylla* var. *yunnanensis* taxonomic rank has been in dispute. This study is about *P. polyphylla* var. *yunnanensis* (‘high stem’), which is described as *P. liiana* sp. nov. It shows significant morphological differences from ‘typical’ *P. polyphylla* var. *yunnanensis*, which include plant height, leaf-blade shape, length, and width, sepal shape, petal color and width, and color of fruit at maturity (Ji et al. 2020). But. they are the same species based on traditional classification. Ji et al. (2020) proposed that *P. liiana* sp. nov might be a new species. Therefore, it is needed to accurately identify this species used the complete chloroplast genome of *P. liiana* sp. nov.

Fresh, healthy, and clean leaf materials of *P. liiana sp*. nov were collected from Gucheng District, Dali, Yunnan, China (25°69′14″'N, 100°16′25″E), the voucher specimen was collected and deposited at the Herbarium of Dali University (20191002B4). The total DNA was extracted using the DNeasy plant mini kit (QIAGEN), and next-generation sequencing was carried out by an Illumina NovaSeq system (Illumina, San Diego, CA, USA). About 7.9 Gb of raw data (52,505,044 reads) were assembled by NOVOPlasty (Park et al. 2019, Fan 2020), and the assembled cp genome was annotated by GeSeq with default sets (Tillich et al. 2017,Yang et al. 2020). The annotated cp genome was submitted to the GenBank with the accession number of MT857225.

The cp genome of *P. liiana* sp. nov was 163,860 bp in length, containing a large single-copy (LSC) region of 84,415 bp, a small single-copy (SSC) region of 12,947 bp, and a pair of inverted repeats (IRs) region of 33,249 bp. The overall GC content was 37.0%. The genome comprises of 135 genes, including 91 protein-coding genes, 37 tRNA genes, and 4 rRNA genes. To confirm the phylogenetic position of *P. liiana* sp. nov, a total of 28 cp genome sequences were downloaded from the NCBI database. After using MAFFT V.7.149 for aligning (Kazutaka and Standley 2013), jModelTest v.2.1.7 (Darriba et al. 2012) was used to determine GTR + I + G as the best-fitting model for the chloroplast genomes. Then, Bayesian inference (BI) tree was performed by MrBayes v.3.2.6 (Fredrik et al. 2012), with *Trillium tschonoskii* (MF614015), *Trillium camschatcense* (NC_046451) and *Trillium govanianum* (NC_044638) as outgroups. The results of tree-building showed that *P. liiana* sp. nov was sister to *P. polyphylla* var. *yunnanensis*. It indicated that they possessed close phylogenetic relationships with each other (Figure 1). However, Ji et al. (2020) proposed that *P. liiana* sp. nov may be a new species (Ji et al. 2020). An in-depth study were needed to accurately verify this species with increased sampling of *P. liiana* sp. nov and *P. polyphylla* var. *yunnanensis.* The cp genome sequence of *P. liiana* sp. nov reported in this study may provide useful resources for the taxonomy and phylogeny of *Paris* genus.

**Figure 1.**
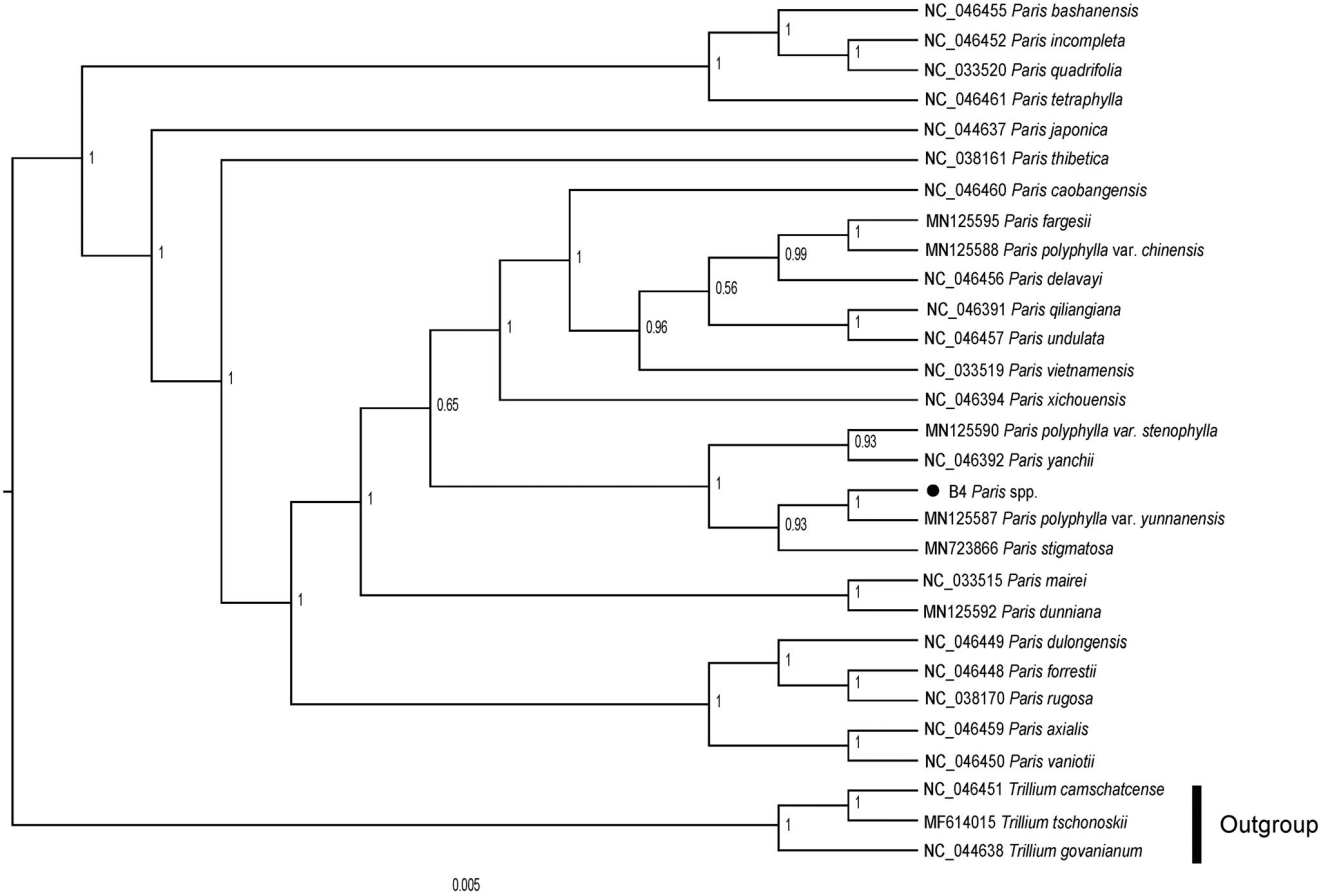
Phylogenetic analysis of 25 species and three taxa as outgroups based on chloroplast genome sequences by bayesian inference (BI) tree, bootstrap support value near the branch.

## Data Availability

The data that support the findings of this study are openly available in NCBI GenBank database at (https://www.ncbi.nlm.nih.gov/nuccore/MT857225) with the accession number is MT857225, which permits unrestricted use, distribution, and reproduction in any medium, provided the original work is properly cited.
